# Supplementary material for: Circulating Pro- and Anti-Inflammatory Metabolites and Its Potential Role in Rheumatoid Arthritis Pathogenesis
Source: Cells. 2020 Mar 30;9(4):827. doi: 10.3390/cells9040827 (PMC7226773; doi:10.3390/cells9040827)
Supplement: Supplementary file 1 [file cells-09-00827-s001.pdf]

## Supplementary Materials

**Supplemenatry Table 1.** Oxylipin abbreviations and names

| Oxylipin | Name | Oxylipin | Name | Oxylipin | Name |
|---|---|---|---|---|---|
| TxB2 | Thromboxane B2 | 20 HDoHE | 20-hydroxy-docosahexaenoic acid | 12-HETE | 12-hydroxy-eicosatetraenoic acid |
| PGF2a | Prostaglandin F2 alpha | LTB4 | Leukotriene B4 | 12-HEPE | 12-hydroxy-eicosapentaenoic acid |
| PGE2 | Prostaglandin E2 | 5,6-diHETE | 5,6-dyhydroxy-eicosatetraenoic acid | 14 HDoHE | 14-hydroxy-docosahexaenoic acid |
| PGD2 | Prostaglandin D2 | 5-HETE | 5-hydroxy-eicosatetraenoic acid | 11 HDoHE | 11-hydroxy-docosahexaenoic acid |
| PGE1 | Prostaglandin E1 | 5-HEPE | 5-hydroxy-eicosapentaenoic acid | 9-HODE | 9-hydroxy-octadecadienoic acid |
| PGD1 | Prostaglandin D1 | 7 HDoHE | 7-hydroxy-docosahexaenoic acid | HXB3 | Hepoxilin B3 |
| PGD3 | Prostaglandin D3 | 4 HDoHE | 4-hydroxy-docosahexaenoic acid | 5-oxoETE | 5-oxo-eicosatetraenoic acid |
| 6k PGE1 | 6-keto Prostaglandin E1 | 9-HOTrE | 9-hydroxy-octadecatrienoic acid | 12-oxoETE | 12-oxo-eicosatetraenoic acid |
| dh PGF2a | 13,14-Dihydro-prostaglandin F2alpha | 5-HETrE | 5-hydroxy-eicosatrienoic acid | 15-oxoETE | 15-oxo-eicosatetraenoic acid |
| dhk PGF2a | 13,14-dihydro-15-keto-prostaglandin F2a | 5,15-diHETE | 5,15-dihydroxy-eicosatetraenoic acid | 9-oxoODE | 9-oxo-octadecadienoic acid |
| dhk PGE2 | 13,14-dihydro-15-keto-prostaglandin E2 | 6R-LXA4 | 6-R Lipoxin A4 | 13-oxoODE | 13-oxo-octadecadienoic acid |
| dhk PGD2 | 13,14-dihydro-15-keto-prostaglandin D2 | 6S-LXA4 | 6-S Lipoxin A4 | 16-HETE | 16-hydroxy-eicosatetraenoic acid |
| bicyclo PGE2 | Bicyclo Prostaglandin E2 | LXB4 | Lipoxin B4 | 18-HEPE | 18-hydroxy-eicosapentanoic aicd |
| 11b dhk PGF2a | 11beta 13,14-dihydro-15-keto-prostaglandin F2a | Resolvin E1 | Resolvin E1 | 5,6-EET | 5,6-epoxy-eicosatrienoic acid |
| tetranor-PGFM | Tetranor-Prostaglandin F Metabolite | Resolvin D1 | Resolvin D1 | 8,9-EET | 8,9-epoxy-eicosatrienoic acid |
| tetranor 12-HETE | Tetranor 12 hydroxy-eicosatetraenoic acid | 7,17 dHDPA | 7,17 dihydroxy-docosapentaenoic aicd | 14,15-EET | 14,15-epoxy-eicosatrienoic acid |
| 11b PGE2 | 11 beta Prostaglandin E2 | 15t-Protectin D1 | 15t-Protectin D1 | 16(17) EpDPE | 16(17) epoxy-docosapentaenoic acid |
| 12-HHTrE | 12-hydroxy-heptadecatrienoic acid | PDX | Protectin DX | 19,20 DiHDPA | 19,20 dihydroxy-docosapentaenoic acid |
| 11-HETE | 11 Hydroxy- eicosatetraenoic acid | 8,15-diHETE | 8,15 dihydroxy-eicosatetraenoic acid | 9,10 EpOME | 9,10-epoxy-octadecenoic acid |
| 11-HEPE' | 11-hydroxy-eicosapentaenoic acidd | 15-HETE | 15-hydroxy-eicosatatrenoic acid | 12,13 EpOME | 12,13-epoxy-octadecenoic acid |
| 13 HDoHE | 13-hydroxy-docosahexaenoic acid | 15-HEPE | 15-hydroxy-eicosapentaenoic acid | 5,6-diHETrE | 5,6-dihydroxy-eicosatrienoic acid |
| PGB2 | Prostaglandin B2 | 17 HDoHE | 17-hydroxy-docosahexaenoic acid | 8,9-diHETrE | 8,9-dihydroxy-eicosatrienoic acid |
| PGJ2 | Prostaglandin J2 | 13-HODE | 13-hydroxy-octadecadienoic acid | 14,15-diHETrE | 14,15-dihidroxy-eicosatrienoic acid |
| 15d PGD2 | 15-deoxy-Prostaglandin D2 | 13-HOTrE | 13-hydroxy-octadecatrienoic acid | 9,10 diHOME | 9,10-dihydroxy-octadecenoic acid |
| 5-iso PGF2a VI | 5-iso Prostaglandin F2 alpha VI | 13-HOTrE(y) | 13-hydroxy-octadecatrienoic acid(y) | 12,13 diHOME | 12,13-dihydroxy-octadecenoic acid |
| 8-iso PGF2a III | 8-iso Prostaglandin F2 alpha III | 15-HETrE | 15-hydroxy-eicosatrienoic acid | Free AA | Free Arachidonic Acid |
| 9-HETE | 9-hydroxy-eicosatetraenoic acid | 8-HETE | 8-hydroxy-eicosatetraenoic acid | Free Adrenic Acid | Free Adrenic Acid |
| 9-HEPE | 9-hydroxy-eicosapentaenoic acid | 8-HEPE | 8-hydroxy-eicosapentaenoic acid | Free EPA | Free Eicosapentaenoic Acid |
| 8 HDoHE | 8-hydroxy-docosahexaenoic acid | 10 HDoHE | 10-hydroxy-docosahexaenoic acid | Free DHA | Free Docosapentanoic acid |
| 16 HDoHE | 16-hydroxy-docosahexaenoic acid | 8-HETrE | 8-hydroxy-eicosatrienoic acid | | |
